# I’m Skinny, I’m Worth More: Fashion Models’ Experiences of Aesthetic Labor and Its Impact on Body Image and Eating Behaviors

**DOI:** 10.1177/10497323221141629

**Published:** 2022-12-07

**Authors:** Alison Fixsen, Magdalena Bailey, Aurore Bardey

**Affiliations:** 1 Department of Psychology, 164128 University of Westminster, London, UK; 2 Burgundy School of Business, 439716 Université Bourgogne Franche-Comté, Besancon, France

**Keywords:** aesthetic labor, eating disorders, modeling, body image, interpretative phenomenological analysis

## Abstract

The fashion industry has been critiqued for promoting ultra-thin bodies, yet the relationship between models’ aesthetic labor and eating disorder (ED) development is unclear. Using interpretive phenomenological analysis, we explored the lived experiences of nine female fashion models including metaphors they used to describe body perceptions and eating behaviors. Four superordinate themes emerged: Shaped for the industry; The body as a market product; Food restriction (“it’s almost glamorized”); Toward a healthier modelhood. Models’ career trajectories were those of lost childhoods, punitive body rules, inadequate dietary advice, and self-regulated food restriction. Models were “shaped” by agents from an early age to conform to the industry’s body rules irrespective of the physiological and psychological consequences. A “toxic” side to this aesthetic industry was depicted; agents were judged callous and money-focused, while idioms like, “feeling like a piece of meat” and “being a hanger of clothes” conveyed a deep sense of degradation and objectification. Ideas instilled at a formative age continued to influence self-image and eating patterns into maturity, pointing to an industrial element to the construction of eating disorders. Our study highlights how infantilization, sexism, and other unethical elements become normalized in poorly regulated industries such as fashion, with dire consequences for the health and wellbeing of employees. Model agencies should recognize the impact of occupational edicts and poor communication on young recruits in a sensitive phase of personality development. Finally, we advocate for more acknowledgment and further investigation into eating disorder construction commercial/industrial side.

## Introduction

Female fashion models can be said to exemplify women’s struggle to conform to aesthetic codes surrounding female sexuality and the tyrannies of age and thinness (Barkty, 1988; Lewak & Ridley, 2017). This highly lucrative industry’s^1^ portrayal of youthful leanness, along with the lack of regulation overseeing this aesthetic labor market, has propelled research toward exploring fashion’s “toxic” underbelly, including its link with eating disorders (Rodgers et al., 2021). The pressures on women in general to conform to particular body trends have been widely studied, including through objectification theory, wherein external objectification and sexualization of women leads to “habitual body monitoring, which in turn can increase women’s opportunity for shame and anxiety” (Fredrickson & Roberts, 1997: p. 173). This concept has been borne out in studies in which self-objectification has been linked to emotional distress (Neziroglu et al., 2008) and body-focused anxiety (Halliwell & Dittmar, 2004). Online platforms and images now provide a fertile field for body objectification that easily spans ages, genders, and ethnicities (Cheney, 2011; Lazuka, et al., 2020). The fickle nature of consumer interest also means that models must continually reinvent and reshape their bodies, including to a more toned body shape (Frederick et al., 2022). Social attitudes to body shapes and their impact on body perceptions and eating behaviors in fashion models have been examined from many angles; however, few studies have attempted to interpret what model’s *feel* about their treatment by the industry, through the language and metaphors employed by models themselves. In this article, we explore the lived experiences of a cohort of female fashion models of different nationalities, from induction to relative maturity. Drawing on emotional labor and aesthetic labor literature, we explain why traumatic experiences and strict “body rules” around thinness instilled at a formative age continue to influence body image and eating patterns into maturity, indicating an “industrial” element to the construction of eating disorder (ED)s.

### Fashion and Eating Disorders

The term ED refers to a psychological impairment characterized by body appearance concerns, intensive distress and disturbed eating behaviors (APA, 2020). Along with the six main feeding and EDs recognized in diagnostic systems—anorexia nervosa, bulimia nervosa, binge ED, avoidant-restrictive food intake disorder, pica, and rumination disorder (APA, 2020)—a further category, subthreshold eating disorders (SEDs), has been postulated to encompass the gray area between normal and pathological eating (Wacker & Dolbin-MacNab, 2020). These SEDs can be long-standing and have been associated with clinically significant impairments in functioning, psychiatric disorders, and elevated suicide risk (Flament et al., 2015). Much of the debate around ED development hinges on neurobiological and social attributions, but high on the list of likely social factors is the influence of media images and advertising on perceptions of beauty (Mills et al., 2017). Multiple studies have highlighted an association between exposure to thin ideals stemming from the fashion industry and the desire for a thin physique (Super, Khadaroo & Bardey, 2021; Swami & Szmigielska, 2013), some linking fashion image exposure directly to ED development in young women (Levine & Murnen, 2009). Memorable messages about body size are believed to have profound negative psychological and emotional impacts on body image and personal health (Anderson et al., 2014). Here, body image refers to “a person’s perceptions, thoughts, and feelings about his or her body” (Grogan, 2008: p. 3).

With regard to models themselves, the relationship between fashion work and ED development is less clear. Preti and colleagues (2008) suggest that girls already oriented towards symptoms of EDs may choose the fashion profession, as the industry’s pressure to be thin may be more easily accepted by people predisposed to EDs. Other research postulates that, while models may not demonstrate higher incidences of severe ED pathology than standard populations, partial ED syndromes are more common among fashion models than non-models (Zancu & Enea, 2017). Restricted eating in young females can result in growth impairment and late-onset or disrupted menstruation, so for health reasons should be discouraged. Fashion managers in one study considered that EDs in models stemmed from personal characteristics, not fashion industry requirements, thereby portraying themselves as not responsible for such behaviors (Super, Khadaroo & Bardey, 2021). Conversely, over half of models in a 2017 survey reported being threatened with dismissal if they failed to lose weight, which for many resulted in risky behaviors such as skipping meals, imbibing illicit drugs, use of intravenous drips, and self-induced vomiting (Rodgers et al., 2017).

### Aesthetic Labor and Fashion Models

A model’s physique and appearance are their “tools of the trade,” hence, the relationship between work, body, and identity in models is likely to be fraught (Holla, 2016). The term “aesthetic labor” refers to a type of work that relies on “a supply of embodied capacities and attributes possessed by workers at the point of entry” (Warhurst et al., 2000: p.4). The fashion industry is one of contradiction between the repetitive, exploitative, and wasteful nature of clothes manufacturing and the uniqueness, creativity, and glamor of high-end fashion (Arvidsson et al., 2010; Hoskins, 2014). Fashion models may not work on the shop floor, nevertheless they are subject to difficult, usually casual, working conditions, without the benefit of typical employee protections (such as statutory sick pay, parental leave, and unfair dismissal protection), leaving them open to abuse and exploitation (Baard et al., 2004; Hoskins, 2014). Hochschild’s study of emotional labor emphasized the personal cost to employees of constantly “putting on a face” in order to please managers and customers (Hochschild, 1979). Studies suggest that models engage in a good deal of emotional labor to sell themselves and charm agents and clients (Mears & Finlay, 2005). However, unlike most employment, fashion models are also subject to rigid body rules and sexualized labor (Warhurst & Nickson, 2009). Competition in the fashion world has been described as “brutal,” with models going to extreme lengths to get cast in shows (Lewak & Ridley, 2017; Rodgers et al., 2021). Behind the glamor of catwalks, real-life model stories portray the dark and “toxic” side to this industry that includes sexual harassment, self-starvation, drug addiction, and suicidal feelings (Clarke, 2022; Dauxerre & Peronnet, 2017; Helmore, 2017).

Yet modeling remains a highly sought-after profession. Financial rewards at the top of the profession are high and even for lower-paid models there are rewards and compensations, such as photoshoots and travel opportunities. Over time, the constant scrutiny and objectification fashion models face can become a routine (Mears, 2008). In Carr and Mercer’s (2017) phenomenological study, young models found modeling life to be all-consuming, but it also acted as a catalyst for transition into adulthood and could booster self-esteem and confidence. Mears and Finlay (2005) point out that, unlike Hochschild’s female customer-facing participants, models purposely seek to use their body capital; the satisfaction they gain from doing so counterbalances the seedier sides of the job. In Holla’s (2016) ethnography, models made great efforts to maintain a coherent sense of self through acting out different types of “good modelhood,” which served to legitimize their work. The complexity of these findings suggests further work is required to fully grasp modelhood from models’ perspectives (Wallenberg & Thanem, 2017). Our article addresses the following: how models depict their career trajectories, what metaphors are used to describe body perceptions and eating behaviors, and what models’ lived experiences tell us about the “industrial” side of ED development.

## Methods

An interpretative phenomenological approach (IPA) was used to provide conceptual background and guide the study (Pearce et al., 2014; Smith et al., 2009; Smith, 2017). A key component of IPA method that aligns with our own aims in this study is committing oneself to examining, “how people make sense of their major life experiences” (Smith et al., 2009: p.1). In our case, this refers to participants’ thoughts and feelings about initial and subsequent experiences of modeling, and the impact of these experiences on body shape perceptions and eating behaviors. We focused on metaphors used in interviews, believing that metaphor memorably captures the essence of an experience, invokes meanings and representations more vividly than ordinary language, and allows painful memories and feelings to be expressed more safely (Shinebourne & Smith, 2010; Fixsen & Ridge, 2017). Central to IPA is the double hermeneutic, whereby the participant interprets their experience within the research interaction, while the researcher interprets the result through the analytic work (Smith et al., 2009). Our study involved using a multi-vocal approach to analysis, which, to ensure all our voices and experiences were accounted for, led us to view this work in an idiographic yet integrated manner (Montague et al., 2020).

The topic of models’ experiences and their eating behaviors was chosen on the basis of the first author’s in-depth study of eating attitudes and behaviours in members of different communities, the second author’s personal experiences of working in the industry and the third author’s expertise in applying psychological concepts in a fashion context, and the third author’s personal experiences of working in the industry. Each author was able to bring something different and unique to the study, while working together allowed us to find a balance between subjectivity and impartiality.

Participants were selected using convenience and snowball sampling. Study inclusion criteria were female gender, 24+ years of age, and 5+ years of experience working as a professional fashion model. Non-English speakers and those currently undergoing treatment for an ED were excluded. The final sample consisted of nine female models of mixed nationalities (Poland, United Kingdom, Russia, Ukraine, Australia, and Denmark), which aligns with the homogeneity and sample size recommended by Smith (Smith et al., 2009). Participants had an average age of 25 years, with 7 years of experience. A semi-structured interview guide was co-produced by the first two authors. Questions focused on capturing strongly felt experiences of being a model, the perceived effects of modeling life on social life and eating practices, participants’ stance in relation to these events (for example, feeling traumatized, not able to speak up), and how this influenced their perceptions of self and industry. Flexible interview questions and interview format were used to allow participants to speak broadly about their experiences (see Supplementary File: Interview Guide)*.*

### Data Analysis

Data analysis took place in two phases. Phase one involved author two thoroughly familiarizing herself with each transcript, then studying the data set in its entirety. Author two then sought out patterns between participants’ views and perceptions of the modeling and themselves and shared these with author one. In Phase 2, a secondary analysis of data was undertaken by authors one and two, in discussion with author three. At this stage, the authors adopted a broader lens, to reflect the stories of participants back to an essential experience and locate these experiences within a wider socioeconomic context. In particular, we sought to interpret experiences shared with others in similar situations (in terms of age, work sector etc.) and factors that appeared unique to models. In addition, we employed psychological and social theory to understand and interpret terms and metaphors used by participants (such as around objectification and aesthetic labor).

Each transcript was read and coded by the authors independently, highlighting descriptive words and phrases and conducting a systematic and critical reading of the transcript to identify developing themes in the text. Following a team discussion, major themes were grouped into clusters, and participants’ quotations were selected. Finally, all authors reviewed the superordinate and subordinate themes to check that participants’ experiences and feelings were clearly and authentically represented in the analysis. To retain the sense of transition expressed by participants, we organized the superordinate themes chronologically, starting with early experiences of models, the development of their careers, changes in views and perception, finishing with reflections on changes in themselves and in the industry. All authors collaborated on the various drafts on the paper. Through rigorous analysis, we identified themes of high prevalence and depth of response in terms of the group’s collective experience.

### Ethical Considerations

A university research ethics committee provided ethical approval for this study. All participants were fully informed about the study and provided written consent prior to interviews. Excluding anyone under current treatment for an ED aimed to filter out more vulnerable participants who might require medical advice. Even so, interviews can trigger concerns and memories, hence, all participants were provided with a debriefing sheet post-interview, which included details of ED support organizations. All interviews are fully anonymized, and pseudonyms and identifiers have been omitted.

## Findings

Our data yielded vivid, insightful accounts of participants’ experiences of the modeling world, including their passage from novice to experienced model. Prominent in these stories were the topics presented under four superordinate themes below: Shaped for the industry (seven participants); The body as a market product (eight participants); Food restriction—“It’s almost glamorized” (seven participants); Toward a healthier modelhood (six participants).

### Shaped for the Industry

Participants vividly recalled their initial induction into the modeling industry. While some had come to modeling in their late teens, others spoke about early aspirations; “When I was 13 I wanted to be a model and my mum understood that it just won’t go away.” Some knew nothing of modeling until scouted by agents; one model recounted the shock and intimidation she experienced during a lightening transition from a sheltered rural childhood to the metropolis:

I was scouted very young. It was on the school trip. Where I am from nobody is really in fashion industry, so I didn’t know anything about it. I had a very easy upbringing and nobody I knew was working in the fashion industry. So, I was quite intimidated, quite scared…

One model recognized her experience of pubescent scouting as far from unique; she knew “loads of girls from the same agency” that had been scouted very young, and to some extent this lessened her sense of aloneness. Fears had also been tempered by a real sense of excitement and anticipation at this time, as the modeling world offered glimpses into a fantasy existence such as traveling to big cities like New York and Tokyo, and other promotional and social opportunities.

Participants had soon discovered a darker side to modeling. Lacking prior knowledge of the industry and its tough demands, they had learned about it the hard way—through observation and personal experience. Fashion industry culture turned out to be one of disregard and disposability; girls working for big agencies received little support, while bookers turned out to be two faced; “They pretend they have your best interest at heart but… they just turn on you and it can be very nasty.” Growing up for young models came to be understood as looking and acting older and more confident than you felt inside, as suggested by this model’s story:

I’m being treated as a professional working model at the age of 13. And you know you’re expected to be able to work, able to communicate with adults at such a young age and also you’ve got that whole thing of… your value being judged on how you look like and I think… as a young woman that can be very tough.

A universal realization was that to remain of interest to modeling agencies, and crucially, to find one’s way on to the catwalk as a “runway model,” one had to be super skinny. Early on, participants had become aware of the hierarchy between commercial and high fashion or runways models (the latter being higher wage and status). As a result, losing weight was treated as *the* key achievement and led to praise and congratulations from agents. Even at a young age, participants had recognized that these edicts were unhealthy; “Looking my slimmest and hearing that I have never looked better, it just felt wrong.” One model spoke of how she was scouted as a 14-year-old girl when she was “a stick” and the agency had not liked it when she had got “curvier” as she went through puberty; “It’s not fair because you were scouted as a child,” she declared.

It was not only physical maturation that was heavily modulated by the job requirements; working in a highly competitive industry at an early age, most models had been forced to prioritize things differently to their peers at home or university. Participants spoke of girls from “other countries” who were under pressure to send money back to their families, but even for those with parental support, the unpredictability of modeling life made it extremely difficult to plan ahead; “You can’t really plan because you don’t even know what you will be doing tomorrow.” One participant spoke about emotional stress on the entire family and of seeing close childhood relationships “fall apart.” As in Carr and Mercer’s (2017) study, some participants viewed this enforced maturity as a double-edged sword; one participant acknowledged that her precocious beginning had given her social confidence but concluded that, “we [models] sacrifice a lot more than people working in other industries.”

### The Body as a Market Product

By and large, participants came to perceive themselves as judged primarily on their body measurements, which created ongoing-anxieties, for example,“[You have] this fear hanging over you that someone will check it on you, and wants to measure you all the time and you’re really under someone else's control.” For runway models, expectations around body measurements were very strict. Participants spoke of the “stigma” they had felt at having a waist more than 25 inches, which in hindsight seemed, “crazy− because these measurements are tiny.” This sense of being constantly judged had, for some, resulted in an excessive degree of body consciousness even in mundane situations, as this model explained; “When I’m walking down the street, it is just subconscious. I would feel that people are constantly judging me and that is going through my head.” Appearance anxieties were exacerbated by veiled hints from agents concerning their overall look; “Like, what’s *‘being expensive’* supposed to mean? Should I go and buy Gucci clothes or what am I supposed to do?”

Body comparisons were most evident during castings, where participants were competing for a job with other models. Recognizing that when, “I’m skinny, I’m worth more,” a model explained how she had increased her exercise regime, dieted, and avoided social events. In turn, she had been congratulated for losing a significant amount of weight by the modeling agency. Some had seen the unhappy consequences for models who did not conform to the required measurements. Those who gained weight within a 1-week period could be punished:

In China every week you had to go to the agency because they would check your weight and measurements. And it didn’t happen to me, because I was 15, I didn’t gain any weight, but for some girls they wouldn’t give them pocket money, if measurements would be one cm bigger. And this money is basically for living.

However, even a slight weight gain could cause a significant change in the agency’s attitude towards a model, participants pointed to the lack of consequences for models who were clearly underweight, with no clear boundaries drawn about how low one’s measurements should fall; “They don’t have a problem…if you’re too skinny.” At the same time, a model’s body shape was also expected to be malleable to the requirements of a particular show or season, which added to the sense of frustration and rejection when body rules changed:

I was told that I was first too big and then too skinny, and it is just constantly… I found it so hard. They just sent me home knowing that I have a problem, they didn’t tell me how to deal with that. Like, “*you’re too skinny you’re in, you’re too big you’re out.”*

All participants described feeling highly objectified both by model agencies and clients, even while desiring to be valued as “more than just the face.” Rather than being respected as an individual with thoughts and feelings, one model felt like a “hanger for clothes.” This participant’s perception of the ideal model from the industry’s perspective strongly evokes Foucault’s archetype of the “docile body” as inapt and pliable (Foucault, 1977): “You’re treated like an object a lot of the time. Then people expect you to be passive and just not to have an opinion on anything.” Not only did models feel externally objectified, they came to measure their own worth in terms of its market value; “So, your whole existence is about your job…you just start thinking about yourself as a product.” This sense of being treated as a product could feel like a personal attack on one’s identity, especially as agencies appeared to show little concern for a model’s emotional stability, safety or ability to fund basic needs. Here one participant describes her feelings of humiliation about being sent off for shoots at the whim of her agents, as if she were a carcass rather than a human being:

Basically, I felt like a piece of meat… whoever wanted me they just sent me there without a reason. ‘*If you got a job, that’s great, but if you didn’t, not my problem*.’ Just like this…when you are like 16 you are not prepared for this kind of thing.

The result was that a model’s body could become a battlefield, and a site of struggle between natural (female) physiology, rules and preferences of model agencies and clients, and the model’s sometimes desperate search for ways to achieve smaller measurements and/or look sexy, just to be able to work and survive in some foreign city.

### Food Restriction—“it’s almost glamorized”

A consequence of the industry’s focus on the skinny body was that participants came to regard food restriction as an essential component of the job. The social stereotype is of models as naturally skinny, however, in participants’ experience many models struggled with their weight and were forced to go to extreme lengths to achieve and maintain the desired body shape; “They have to ruin their bodies to look like that.” Participants spoke about their early fears with regard to rules around, “the right body shape.” Some had experienced a strong sense of fear and rejection; “you go into puberty and grow and get curvier, agencies are not happy about it.” No participants spoke of active EDs, however, some had experienced bulimic episodes, while others had witnessed how potential EDs could be ignored by agents, who seemed most interested in keeping a model working, as this participant explained:

As long as you make them money, I don’t think they really care if you’re healthy or not. And I had experiences that I told someone from the agency, *“I think one girl has a problem…because I could see her throwing up.*” And they would just say, “*we will talk to her,*” but then that’s it…they kept working these girls in town.

While recognizing such behavior as being unhealthy, eating as little as possible had at some point become a life goal in itself, to the extent that it took over models’ lives. Three of the participants had experimented with a vegan diet for weight-loss reasons and for two this had marked the beginning of further and stricter restrictions. This participant recalled her gradual progression:

So, I started not eating meat and then […] I couldn’t go to the restaurants anymore. I started counting calories. I was writing down everything I ate during the day. I don’t know why. I couldn’t even eat salad in a restaurant because I didn’t know the exact amount of oil in it… I was eating some rice sometimes with a piece of cheese on it…If I got less than 1,200 calories that felt amazing.

The lengths that models were willing to do to fit into the high fashion category was described as “scary.” During big shows, models would eat virtually nothing, exercise excessively and never rest; “There is no time to eat or they eat super clean and its almost glamorized, especially during the fashion week.” Things were slowly changing with such things as new medical checks to ensure “very ill girls” were turned away from shows, but agents were slow to react. In the absence of clear advice, information was sought through Internet sources, however, for the model below, it resulted in such excessive weight loss that she was sent packing by the agency:

I was trying to do everything on my own. I was watching YouTube videos of how to stop eating and reading about ways of reducing calories, diets… Then I was extremely skinny, and they sent me home.

### Toward a Healthier Modelhood

Although participants depicted their early modeling days as traumatic, these models had chosen to remain in their trade and had learned to make sense of early trauma. One participant, who shared, “being traumatized so many times,” had gone on to realize that much of this trauma related to the industry’s obsession with underweight models. Having learned the harder realities of modelhood, it was hard for these more veteran models to witness the starry-eyed optimism of new recruits concerning modelhood: “They [the young recruits]…thought it will be easy and everyone will love them and it’s not like that.” The experience of casting, for example, had its benefits in terms of learning how to manage the criticism, however, as this participant explained, the constant rejection was ultimately soul-destroying:

If you’re going for a casting you may be rejected nine times out of ten, or even ten out of ten. On the one hand, it is good because you learn how to… take it. Or, how to not take it personally because it’s not because of you… but on the other hand if you don’t understand that it is not because of you… you destroy yourself- you feel like a failure and it’s tough.

Over time, participants had found ways to cope with the work demands, and most had settled into a pattern of life which felt more comfortable. A big part of this was learning to stay resilient in the face of ongoing judgment and frequent rejection. As this model remarked; “It took me a few years to gain that thick skin.” Yet, participants remained indignant about the lack of sound advice and empathy from employers within the modeling industry. This model felt that agencies needed to be more cautious and sensitive when evaluating a model’s appearance, and stressed how criticism can affect models in a long run:

[…] Also if you don’t take critique too well, I think it may affect you a lot. Critique from agencies especially. Bookers sometimes can’t use nice words to tell you to lose weight, they just tell you straight forward like “*hey, you’re too big!”* even if they treat it as some joke, girls think that… it can just really affect them and then you keep thinking about your body in a bad way.

Having experienced the detrimental effects of its strict body requirements, most participants were keen to see changes in beauty standards in the industry; “More girls of all sizes on catwalks would really make a difference.” This advice applied in particular to very young girls, as it was, “a lot of easier for them to get sucked into this toxic way of thinking.”

The desired look on social media had become healthier, and cursory moves in modeling industry toward diversity were afoot, however, participants did not consider these changes to reach far, as most agents continued to hire models who were ultra-thin; “If you’re doing a swimwear, lingerie that’s ok, but most fashion catwalks, high fashion editorials still like the thinner the better.” In addition, some participants admitted to maintaining this preference for body thinness which been drilled into them over years; “I’m trying not to judge because I think my perception is somehow screwed.”

Participants emphasized the moral requirement of the industry to safeguard young people who want to pursue a modeling career; “No one really knows what is happening behind the closed doors, no one really sees what goes on.” To prepare for the job, participants strongly recommended that new recruits be properly briefed about the realities of the industry, so they are better equipped to cope with the challenges than they had been. Their hope was that new girls would be better prepared to face the vicissitudes of modeling life; “I wasn’t looking after myself properly; if there is a new girl coming in, I would want them to be aware of the realistic side of it.”

## Discussion

Our overall interpretation of the data is that “modelhood” is best conceptualized as a passage or journey, which for participants was characterized by emotional trauma, homesickness, and inflexible rules defining weight and measurements, that led on to self-regulated food restriction. For those whose model career started very early, job demands replaced usual childhood pursuits, leading to a sense of a lost era for some. A brutal side of the industry was reported, with young female models who failed to attain the physique required punished and potentially left destitute in a foreign country. Studies suggest that even isolated negative remarks about weight are remembered many years later and retain negative body associations (Anderson et al., 2014). These formative experiences could create a mindset wherein a model’s sense of worth and safety became intrinsically bound up with achieving thinness which, a priori, carried through into eating behaviors and food choices. Those who started modeling later had a more robust perspective in terms of priorities, reaction to criticism, and general copying strategies. Even these women depict modelhood as physically and emotionally consuming, while fears about body shape and weight gain had proved hard to shift. For the rest of our discussion, we bore down into the nature of “modelhood,” its association with EDs and finally, consider what can be done to create a more ethical, power-balanced work environment for models.

### Metaphors of Modeling

The word “model” has several meanings (Oxford Learner Dictionaries, 2022). First, model means a *representation of a person or thing*. That negative messages concerning body size and image are frequently more memorable than positive messages (Anderson et al., 2014) which was borne out by the strong metaphors study participants used to depict their effects. Phrases such as, “being treated like an object” and “feeling like a product,” were used to express an overwhelming sense of objectification within the industry. The expression, a “hanger of clothes” suggests a shapeless body, with no or few visible curves disturbing the line of the garment. The youthful slender body remains virtually mandatory in high-profit, high-end modeling (Holla, 2016). In addition to the physiological consequences of a low body mass, this pre-pubescent look has served to exclude black girls and women from aesthetic professions such as modeling and ballet (Robinson, 2021). The idiom, “feeling like a piece of meat” has carnal connotations; in urban slang, it implies, “reducing a woman down to her sexual parts”(dictionary.com, 2022). A climate of tolerance to some forms of sexual harassment can exist in certain organizations, which may be perceived as benign, but nevertheless have damaging consequences for employees (Buchanan et al., 2014). Under-age models are known to face a multitude of injustices and are highly susceptible to both financial and sexual exploitation (Paccione, 2016).

The word “model” also means *to fashion or shape in a malleable material* (Oxford Learner Dictionaries, 2022). Participants in this study described how from an early age they were being “shaped” by agents to conform to the industry’s body rules, no matter the physiological and psychological consequences. The idea of the docile body that is shaped or molded for aesthetic and profit reasons has been explored in other gendered sectors such as ballet (Green, 2002). A fashion model’s overall “look” and sexuality is controlled by agents, bookers, designers, and stylists (Holla, 2016), thus, the sense of embodiment described in studies of dancers (Green, 2002; Robinson, 2021) was absent from our study. Models are also so-called because they *set the mode or fashion,* not just for clothes but for body shapes. The fashion sector works through a circle of networks that is fired by inspiration but is ultimately driven by market forces (Amed et al., 2020). High fashion epitomizes glamor and luxury; another remit of models in our study was to “look expensive.” Thinness in western culture symbolizes competence and success (Bogár & Túry, 2019). This myth, combined with other commercial and lifestyle pressures on models, puts them at risk of developing body anxieties and a subclinical or full-blown eating disorder (Preti et al., 2008; Zancu & Enea, 2017).

### From “body rules” to Body Positivity

Our findings indicate that externally imposed “body rules” as exemplified in the fashion industry are as, or more powerful, in their psychological effects as the “feeling rules” (such as smiling sweetly to please customers) described in the established literature on emotional labor (Hochschild, 1979). Lack of embodiment, coupled with the fickleness of the fashion market, means the “aesthetic labor” performed by the average model may feel distinctly alienating (Mears & Finlay, 2005). Attempts to mold themselves to other’s requirements may also influence younger models’ emergent sense of identity, leading to self-doubt and self-devaluation (Baard et al, 2004). This, along with other factors associated with negotiating a labor sector that treats most employees as highly disposable, puts models at high risk of developing unhealthy eating behaviors (Bogár & Túry, 2019; Treasure et al., 2008).

Times are changing; women are being urged to resist the more destructive messages of Western capitalist culture and develop a sense of self-worth beyond appearance or weight status, through personal agency and community involvement (Wacker & Dolbin-MacNab, 2020). More models may come to reject the idea of dieting and opt to pursue their own “healthy” versions of modelhood (Holla, 2016). Developments such the body positivity movement (Lazuka et al., 2020), “metrosexual” fashion (Bogár & Túry, 2019) and transgender models should help to empower members of the modeling community to insist on individual voices being heard, and diverse body shapes being represented. In recent years, more attention has been paid to legislation and policies improving working conditions and protecting fashion models (Rodgers et al., 2017). In the United States and France, legislation now includes private changing areas and medical certificates to protect models from pressures to be extremely thin; however, recent model surveys indicate these interventions have yet to achieve their intended goals (Rodgers et al., 2021). In the meantime, models in many countries remain subject to unfair and sometimes degrading demands of agents and customers. Digital communication channels pose new risks to models, since compromising photos can be shared on social media (Rodgers et al., 2021). Digital platforms have also been linked to new EDs and eating trends including orthorexia nervosa, a pathological fixation with foods which participants referred to in the stories. The muscular female body shape rapidly gaining popularity via the Internet creates new body rules and self-objectification issues for women (Bozsik et al., 2018).

## Conclusions and Limitations

This study illustrates how intransigent body rules, infantilization, sexism, and other unethical elements can become normalized in under-regulated work sectors such as the fashion industry, with dire consequences for the health of their employees. It illuminates the commercial/industrial side of ED construction and the need for policy makers and industries to frequently review industrial equality and harassment policies. Agencies would do well to recognize the deep psychological impact of industry requirements, coupled with lack of nutritional guidance, on girls in a sensitive phase of physical and personality development, and to conduct regular self-assessments of sexual harassment (Buchanan et al., 2014). By focusing on young white female models, our research missed out voices of models of other ages, genders, ethnicities, and physical abilities; further studies should explore experiences of different communities. More research into the “industrial” construction of EDs in other body-focused sectors such as fitness is also advocated. This project’s scope was limited by University course requirements and absence of external funding. Although we were mindful of setting aside our own views and experiences of the subject matter, we are aware of the limits to doing so. Instead, we adopted a self-critical and reflective approach, seeking to understand how our different perspectives and assumptions could help to inform our approach and conclusions. By adopting an interpretative approach and exploring models’ life journeys and experiences, we believe our work provides a unique perspective on aesthetic body rules and their link with dysfunctional eating behaviors, both in models and those seeking to emulate them.

## Supplemental Material

Supplemental Material - I’m Skinny, I’m Worth More: Fashion Models’ Experiences of Aesthetic Labor and Its Impact on Body Image and Eating Behaviors Supplemental Material for I’m Skinny, I’m Worth More: Fashion Models’ Experiences of Aesthetic Labor and Its Impact on Body Image and Eating Behaviors by Alison Fixsen, Magdalena Kossewska and Aurore Bardey in Qualitative Health Research

## Notes

1 In 2019, a $2.5 trillion US dollar revenue from the fashion industry was recorded (Amed et al., 2020).
