# Supplementary material for: I’m Skinny, I’m Worth More: Fashion Models’ Experiences of Aesthetic Labor and Its Impact on Body Image and Eating Behaviors
Source: Qual Health Res. 2022 Dec 7;33(1-2):81–91. doi: 10.1177/10497323221141629 (PMC9827487; doi:10.1177/10497323221141629)
Supplement: Supplemental Material - I’m Skinny, I’m Worth More: Fashion Models’ Experiences of Aesthetic Labor and Its Impact on Body Image and Eating Behaviors [file sj-pdf-1-qhr-10.1177_10497323221141629.pdf]

Supplementary file: Interview questions

1. Could you please tell me something about your journey into the fashion industry?
2. How long have you been working as a fashion model?
   (How would you describe this experience; how do you think that affected your life?)
3. What is expected from fashion models?
4. What are past present attitudes to the body image in the fashion industry?
5. Who guides and supports models during their career?
6. What's your experiences of the attitudes to physical health of fashion models you have been working with during your career?
   (why do you think is that)
7. How common do you think eating disorders are among fashion models?
   (Why do you think is that?)
8. What do you think about a psychological health of fashion models you have been working with?
9. If there is anything, what do you think could improve fashion models' well-being?
10. In your experience, what types of personality traits are seen as desirable by the fashion industry if any?
11. What do you think are society's attitudes towards body image
    (How that affect attitudes towards fashion industry?)
